# Insulation failure mapping on power transformer bushing using FRA and electrostatic simulation

**DOI:** 10.1371/journal.pone.0353356

**Published:** 2026-07-07

**Authors:** Salem Mgammal Al-Ameri, Waleed M. Hamanah, Ali Ahmed Salem, Mohd Fairouz Yousof, Samir Ahmed Al-Gailani, A. Abu-Siada

**Affiliations:** 1 Interdisciplinary Research Center for Sustainable Energy Systems, King Fahd University of Petroleum and Minerals, Dhahran, Saudi Arabia; 2 Department of Electrical Power Engineering, Faculty of Electrical and Electronic Engineering, University Tun Hussein Onn Malaysia, Batu Pahat, Johor, Malaysia; 3 Department of Electrical Engineering, Faculty of Engineering, Aden University, Aden, Yemen; 4 Electrical and Computer Engineering Discipline, Curtin University, Bentley, Western Australia, Australia; Swiss Federal Technology Institute of Lausanne, SWITZERLAND

## Abstract

The power transformer bushing is a critical insulation component and a frequent contributor to transformer failures. This paper presents combined electrical and Multiphysics approaches for mapping failure mechanisms in Resin-Impregnated Paper (RIP) transformer bushings using frequency response analysis (FRA) and electrostatic analysis. An 11 kV/0.433 kV, 500 kVA power transformer bushing is selected as a case study and modeled using a MATLAB-based equivalent circuit to simulate its FRA characteristics. Variations in bushing circuit capacitance are introduced to represent insulation degradation, surface pollution, and insulator damage, and the corresponding failure modes are mapped through their distinctive FRA signatures. To provide physical insight into these failure mechanisms, a detailed model is developed in COMSOL Multiphysics, where degradation, pollution, and destructive damage are analyzed using electric field and electric potential distributions. The results from the FRA simulation and electrical Multiphysics in this paper provide a framework for understanding the behavior of transformer bushing failures and enable early fault detection. This also improves the transformer bushing maintenance and condition monitoring.

## Introduction

Power transformers are one of the prime components used in power systems. The power transformers’ healthy operation is essential to maintain the power system stability and continuity. Power transformer high voltage bushing is one of the various insulation parts that function as an insulation path to the current-carrying conductor through the transformer tank. The power transformer bushings are critical, and they are the cause of major failures of the transformers and unplanned power interruptions [1,2]. The insulation used in transformer bushings is made of oil-impregnated paper (OIP) or resin-impregnated paper (RIP), and graded foil (RIF) is used as a supplement to optimize the electric field distribution. Due to long-term working conditions, bushings will be exposed to electrical stress, thermal stress, and environmental stress, resulting in insulation aging, water emergence, partial discharge, and degradation of dielectric strength [3].

The power transformer bushing insulation degradation is gradual, and detection using traditional maintenance may not be possible before a failure occurs. The traditional transformer bushing condition assessment and diagnostic techniques include capacitance and loss factor (tan δ) measurement, dissolved gas analysis (DGA), and infrared thermal imaging [4]. The withdrawal of using these conventional methods is that they have limited sensitivity to early degradation. In particular, local insulation degradation or small changes in dielectric properties may not cause a sufficiently large change in the measured parameters to trigger an alarm.

The Frequency Response Analysis (FRA) is a recognized tool used to detect the mechanical defects in the power transformer [5]. This corresponds to the electrical inductance and capacitance in the power transformer. This makes it practically possible to identify the insulation condition and related faults. However, FRA has been widely used in transformer windings, and its application in transformer bushing insulation degradation. This has been considered in the literature. In [6], it has been investigated using the FRA method for bushing thermal aged, thus providing an opportunity for early fault detection.

**Fig 1:**
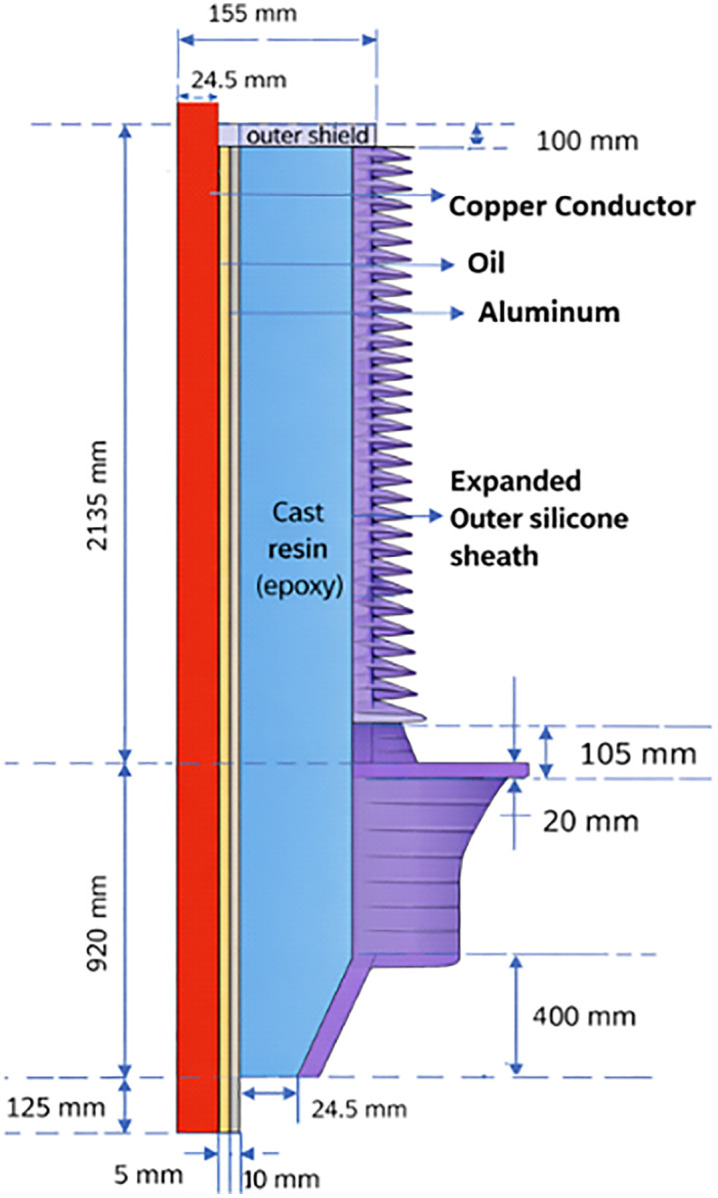
Typical 1ZBF690 series structure of a power transformer bushing, highlighting the main insulation layers, conductor, and grading foils [9].

For investigating the power transformer bushing, the digital twin modelling method became an applicable approach for this study. The electrical circuit equivalent to a power transformer with a bushing can be used to simulate a transformer FRA signature. The results will help to study insulation degradation mechanisms, such as capacitance decreases [7]. Other approaches using Multiphysics simulation platforms, such as COMSOL Multiphysics, can conduct detailed studies on the electric stress distribution and electric potential behavior. The power transformer bushing can be modeled and perform electrostatic analysis, thus providing valuable physical insights into the degradation process [8]. The typical transformer bushing can be modeled using the parameters presented in Fig 1. The transformer bushing core is always made of copper. Then the insulation components are oil, epoxy, and ceramic.

This paper proposes a comprehensive method for transformer bushing insulation faults rated above 10 kV. The combination of electrical analysis modeling and Multiphysics simulation is used. In MATLAB, an equivalent circuit model of the transformer bushing is established. The FRA spectrum is simulated under healthy and different percentage insulation conditions. This is simulated by changing the bushing capacitance. In COMSOL Multiphysics, a detailed bushing model is constructed to analyze the electric field and electric potential performance. Thereby, the visualizing stress concentration and overheating phenomena related to insulation degradation and failures is analyzed. The correlation between FRA characteristic changes and physical degradation mechanisms is established, verifying the ability of FRA to detect bushing insulation faults in their early stages.

This paper can highlight the following contributions in the field of power transformer condition monitoring, particularly in bushing insulation, as follows:

- This study focused on the transformer bushing insulation degradation using the FRA method. This was limited to studying transformer winding deformations.
- The electrostatic method was used to study the electric field distribution. But this study focused on different electrostatic stress behaviors on the surface of transformer bushings.
- This research bridges the electrical-based and electrostatic modelling and simulation to establish a realistic failure mapping framework.

In this study, the physical mechanism governing bushing insulation degradation is attributed to long-term thermal aging and localized thermal stress. Under continuous operating temperatures and localized overheating, the resin-impregnated paper (RIP) or epoxy insulation experiences chemical structural degradation, leading to a reduction in the material’s effective thickness which breakdown of the dielectric properties. This physical degradation presented electrically as a progressive reduction in the bushing’s equivalent circuit capacitance (Chb). By focusing on this decreasing capacitance trajectory, the proposed electrical equivalent circuit and frequency response analysis (FRA) framework can accurately map the specific structural faults and dielectric loss behaviors induced by severe thermal aging and partial discharges, distinguishing them from other environmental degradation pathways.

## Electrical approach analysis

### Circuit model

This method is based on an RLC equivalent circuit representation of a power transformer, including its insulation bushings. Power transformer modelling approaches are generally classified into three categories: black-box, physical (white-box), and hybrid (grey-box) modelling. In this study, a physical modelling approach is adopted, in which the transformer is represented by an RLC equivalent network whose elements correspond to the transformer’s physical structure [10]. Based on this model, the transformer FRA is simulated. The FRA is expressed as a Bode plot of the ratio between the output (U2(f) and input (U1(f) voltages, U_2 (f)/U_1 (f) as given in (1). The Bode magnitude is defined as the logarithmic amplitude, as given in (2).


$$\begin{array}{c} H(f)=\frac{U_{\text{2}}(f)}{U_{\text{1}}(f)} \end{array}$$ (1)

$$\begin{array}{c} K(f_{\text{1}})=\text{2}\text{0}{\text{log}}_{\text{1}\text{0}}\frac{U_{\text{2}}(f)}{U_{\text{1}}(f)} \end{array}$$ (2)

**Fig 2:**
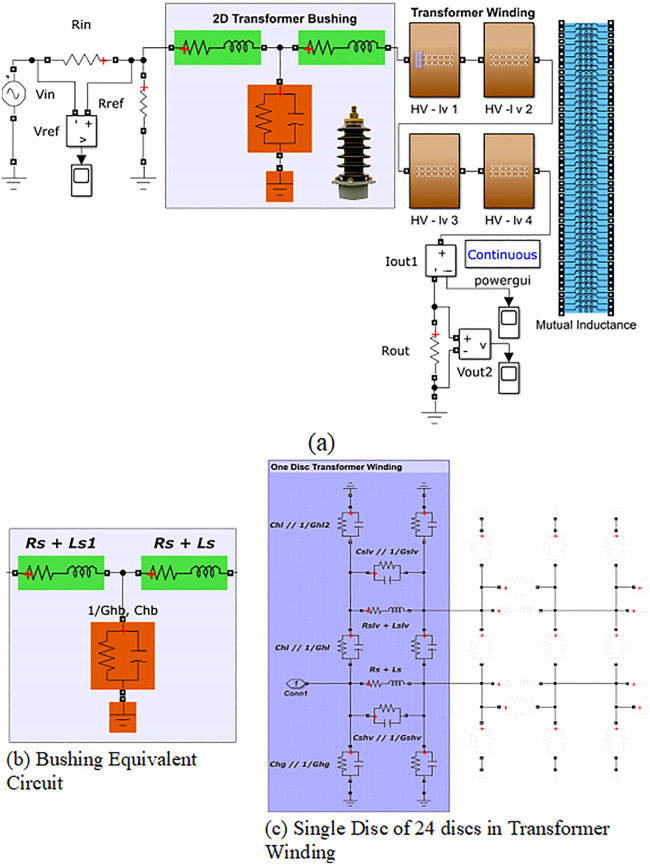
Power transformer modeling (a) overall transformer equivalent circuit model, (b) equivalent transformer bushing model, (c) transformer winding discs.

The equivalent circuit is constructed based on the physical structure of the bushing, as shown in Fig 2. The central copper conductor is surrounded by cast resin epoxy insulation and an external silicone sheath. Capacitive coupling between the high-voltage conductor and ground, as well as between different conductive components of the bushing, is represented by multiple capacitances. These include the high-voltage-to-ground capacitance, high-voltage-to-low-voltage capacitance, and stray capacitances associated with the insulation system and nearby metallic parts. Each capacitance is modelled in parallel with a conductance element to account for dielectric losses arising from insulation conductivity and ageing effects.

**Fig 3:**
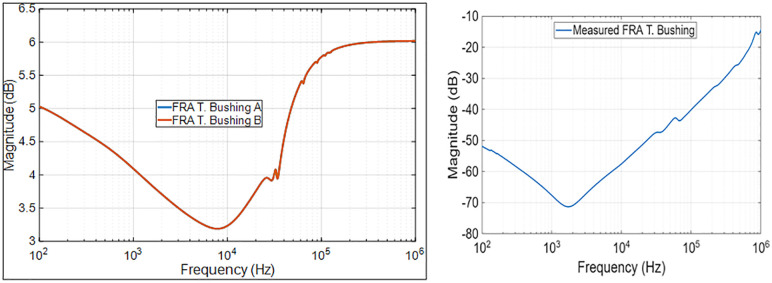
Power Transformer (a) FRA Simulated at healthy bushings (A, B), 0% changes in capacitance (b) compared to the actual measurement from the 11 kV/0.433 kV, 500 kVA transformer.

Series resistances and inductances are incorporated to represent the conductive path resistance and stray inductive effects of the bushing conductor and associated connecting leads [11]. The distribution transformer circuit parameters presented in Table 1 are based on an actual untanked unit transformer rated at 11kV/0.433kV, a 500 kVA, and placed for testing at Tanaga National Berhad (TNB). The transformer’s main parameters are obtained and calculated to simulate its frequency response analysis as in Fig 3(a). This parameter set represents the with a healthy operating condition of the transformer bushing and serves as a reference for subsequent degradation analysis. Insulation degradation is modelled by progressively reducing the capacitance values in the two-dimensional bushing model, corresponding to changes in insulation permittivity. Such capacitance changes reflect insulation ageing mechanisms, including thermal stress, partial degradation of the epoxy resin, and partial discharge activity. In parallel, an increase in conductance is used to represent higher dielectric losses associated with insulation ageing. This modelling approach establishes a direct link between physical degradation mechanisms and measurable electrical response.

**Table 1:** The transformer equivalent circuit parameters, as based on 11 kV/ 0.433 kV, a 500 kVA transformer, including the bushing in a healthy condition.

Parameter Name	symbol	Value	Unit
HV to ground capacitance	*C*_*hg*_	2.50 × 10 ⁻ ⁷	F
HV to ground conductance	*G*_*hg*_	1.00 × 10 ⁻ ⁴	S
HV series capacitance	*C*_*shv*_	2.20 × 10 ⁻ ⁹	F
HV series conductance	*G*_*shv*_	1.43 × 10 ⁻ ⁶	S
HV to low-voltage capacitance	*C*_*hl*_	6.50 × 10 ⁻ ⁸	F
HV to low-voltage conductance	*G*_*hl*_	1.00 × 10 ⁻ ⁴	S
HV series resistance	*R*_*shv*_	10.2	Ω
lv series capacitance	*C*_*slv*_	1.11 × 10 ⁻ ⁹	F
lv series conductance	*G*_*slv*_	1.43 × 10 ⁻ ⁶	S
lv to ground capacitance	*C*_*lg*_	3.70 × 10 ⁻ ¹¹	F
lv to ground conductance	*G*_*lg*_	1 × 10 ⁻ ⁴	S
lv stray inductance	*L*_*shv*_	1.8 × 10 ⁻ ⁴	H
lv series inductance	*L*_*slv*_	6 × 10 ⁻ ⁵	H
lv series resistance	*R*_*slv*_	0.6	Ω
Bushing HV conductance	*G*_*hb*_	1 × 10 ⁻ ⁹	S
**Bushing HV capacitance**	***C***_***hb***_	**1 × 10 ⁻ ¹²**	**F**
Source/load resistance	*R*	50	Ω

### FRA simulation and validation

The FRA signature is represented by the magnitude and phase Bode plots of the transfer function between the input and output terminals [12,13], as defined in (1). In this study, the magnitude response is used, as it provides sufficient information to assess the condition of the power transformer. Under healthy operating conditions (Transformer Bushing A: refers to an equivalent circuit in Fig 2 at a healthy condition, where the insulation parameters correspond to the nominal (baseline) capacitance values 1 × 10 ^−12^ F as in Table 2), the frequency response exhibits a series of resonances and anti-resonance points resulting from the interaction between the circuit inductances and capacitances. These resonant features form a baseline reference for comparison with degraded conditions.

**Table 2:** Transformer bushing capacitance degradation process.

Degradation level	Percentage of degradation	Capacitance value (*C*_*hb*_)
Healthy	0%	1 × 10^−12^ F
Measured capacitance of the actual bushing	0%	1.47 × 10^−12^ F
Early degradation	10%	1.05–1.10 × 10^−12^ F
Moderate degradation	20%	1.10–1.20 × 10^−12^ F
Advanced degradation	30%	1.20–1.30 × 10^−12^ F
Severe fault	50%	1.30–1.50 × 10^−12^ F

Under normal conditions, the FRA response is simulated, as shown in Fig 3, using a bushing capacitance *C_hb* = 1 × 10^-12^ F. The degradation process (Transformer Bushing B: refers to an equivalent circuit in Fig 2 at a degraded/faulty condition, where insulation degradation is represented by changes in capacitance values, as (10%, 20%, 30% and 50% as shown in Table 2). Bushing faults are primarily induced by changes in the capacitance between the conductor and ground. Five degradation levels are considered, ranging from the healthy condition to a severe bushing fault, corresponding to a total capacitance decrease of up to 50%.

The FRA measurement validation is presented in Fig 3. The comparison with the actual FRA measurement from an untanked power transformer. The measured response is obtained for phase A of a power transformer rated at 11 kV/433 V, a 500 kVA using an end−to−end open circuit configuration. The measurement is conducted by injecting the excitation signal at one terminal of phase-A of the HV winding and measuring the response at the other terminal while the LV windings are left open-circuited. The measured response in Fig 3(b) shows resonance and anti-resonance, which can reflect the traces shown in the FRA form simulated in Fig 3(a). Also, the capacitance of the bushing was measured using CPC from Omicron, and the total capacitance measured was 1.47 × 10 − 12 F.

**Fig 4:**
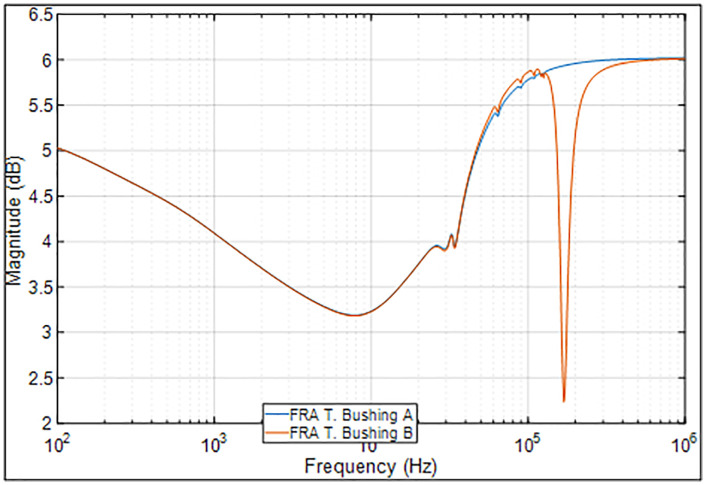
FRA for normal vs very early degradation in transformer bushings.

**Fig 5:**
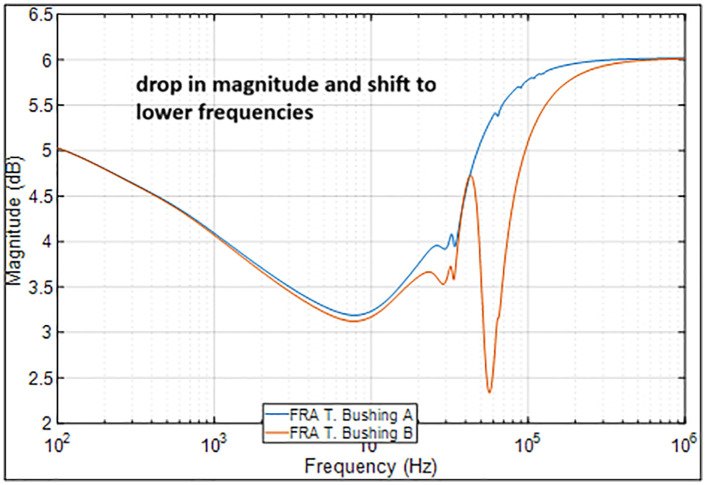
FRA for normal vs moderate degradation in transformer bushings.

**Fig 6:**
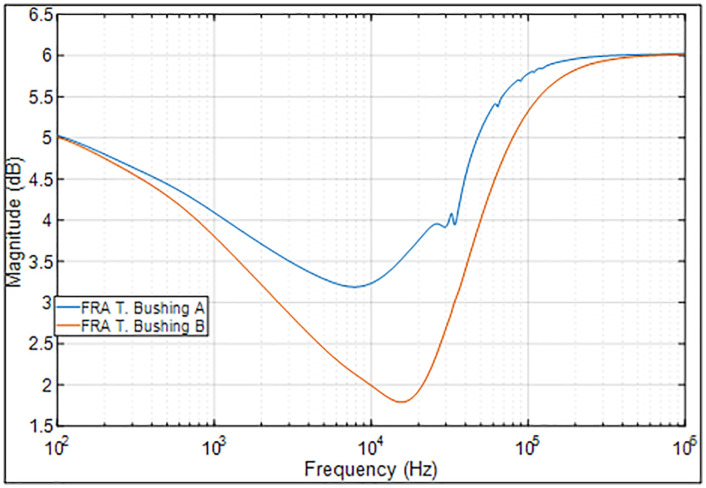
FRA for normal vs advanced degradation in transformer bushings.

**Fig 7:**
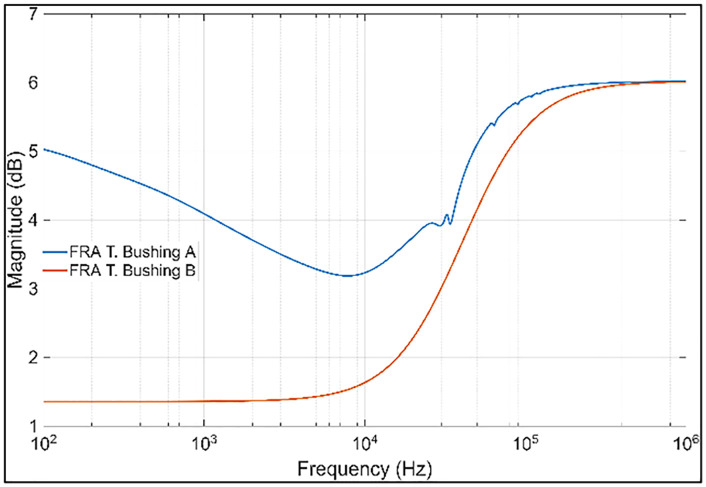
FRA for normal vs severe fault in the transformer bushings.

The FRA results are presented in Fig 3, Fig 4, Fig 5, Fig 6, and Fig 7 illustrates the influence of progressive insulation degradation on the frequency response of the transformer bushing. Fig 3 shows the healthy reference response, characterized by distinct resonance and anti-resonance points resulting from the interaction of inductive and capacitive elements in the equivalent circuit. In transformer bushing insulation degradation, the small capacitance can be reduced to the present insulation degradation. In Fig 4, there is a resonance frequency shift and a magnitude drop due to the small insulation degradation. This is considered the early stage of bushing fault. The FRA resonance variation can become huge and shift towards lower frequencies when the level of insulation degradation increases. The FRA deviation due to minor, advanced, and severe insulation degradation is shown in Fig 5, Fig 6, and Fig 7. The induction of lower dielectric strength causes the FRA signature resonance picks and shifting response to lower frequencies. Under severe degradation conditions, the FRA response exhibits significant magnitude reduction and resonance suppression, suggesting substantial insulation deterioration or fault development. These results confirm that FRA is an effective diagnostic tool for tracking insulation degradation and identifying transformer bushing faults at an early stage, well before catastrophic failure occurs.

The correlation coefficient (CC) quantifies the similarity between two FRA responses, approaching zero for uncorrelated responses and unity for identical responses. In this study, the CC is calculated in accordance with IEEE Std C57.149-2012 and IEC 60076-18 [14,15], using the formulation given in (3).


$$\begin{array}{c} {CC}_{\left(X,Y\right)}=\;\frac{\sum_{i=1}^{N}X\left(i\right)\;\times \;Y\left(i\right)}{\sqrt{\sum_{i=1}^{N}{\left[X\left(i\right)\right]}^{2}\;\times \;\sum_{i=1}^{N}{\left[Y\left(i\right)\right]}^{2}}} \end{array}$$ (3)

According to IEEE Std C57.149 standards [14], CC values greater than 0.98 indicate a healthy condition, values between 0.95 and 0.98 indicate minor deviations, values between 0.90 and 0.95 suggest possible mechanical or insulation changes, and values below 0.90 indicate a high probability of fault. The CC results show that, in the low-frequency region, only severe bushing insulation degradation produces a detectable deviation. Degradation effects initially appear in the high-frequency region and progressively extend toward the mid-frequency range as insulation deterioration increases. As summarized in Table 3, transformer bushing insulation degradation influences the FRA response across all frequency regions. With increasing degradation severity, the response magnitude decreases, starting in the high-frequency range and progressively shifting toward lower frequencies as the bushing capacitance is reduced.

**Table 3:** The correlation coefficient for insulation degradation.

FRA Range	Healthy	Early	Moderate	Advanced	Severe fault
Low-Frequency	1.00	1.00	1.00	0.99	−0.90
Mid-Frequency	1.00	0.98	0.37	0.76	0.83
High-Frequency	1.00	0.08	0.99	0.99	0.99

**Fig 8:**
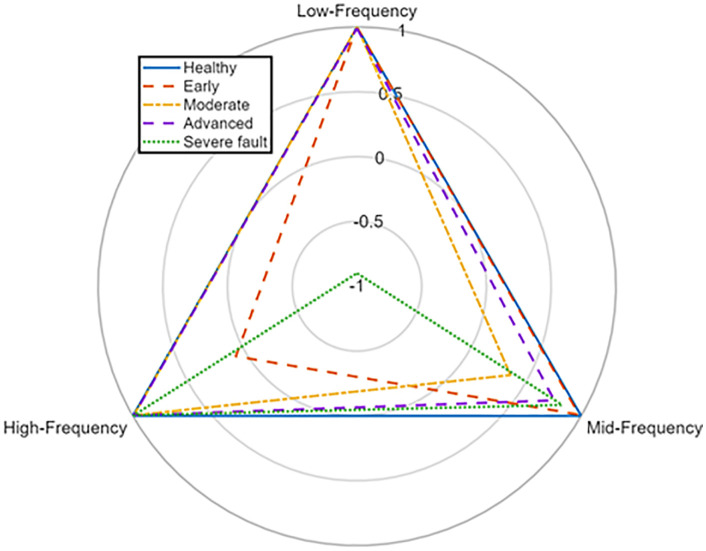
Spider (Radar) Plot of CC for Insulation Degradation.

In this regard, the bushing insulation degradation at each severity level is visualized using a spider (radar) plot, as shown in Fig 8. The healthy condition is represented by a solid line. At the early stage of degradation, noticeable effects appear primarily in the high-frequency region, followed by moderate and advanced degradation influencing the mid-frequency range. Under severe degradation conditions, pronounced deviations are observed in both the low- and mid-frequency regions.

## Multiphysics approach analysis

### Geometrical and materials used

**Fig 9:**
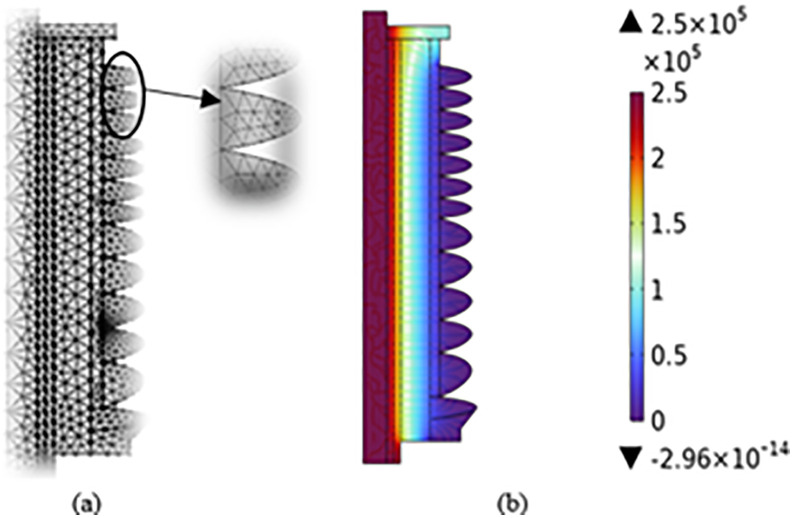
Transformer Bushing Simulation (a) Mesh generation (b) Surface electric potential (V).

The geometry of the transformer winding RIP bushing is designed based on the parameter structure shown in Fig 1 [9]. The selected transformer is rated at 11 kV/0.433 kV, 500 kVA. The COMSOL Multiphysics model, including the mesh design, is illustrated in Fig 9. The bushing insulation consists of three layers surrounding the copper core: transformer oil, epoxy resin, and porcelain. The oil insulation is shielded with aluminum. For this design, the bushing is equipped with 13 sheds. While neither the IEC nor the IEEE standards specified the number of sheds, this parameter is generally chosen based on creepage distance and anti-pollution performance. The design can be extended to 21 sheds if needed. All material properties are selected using the default values provided in COMSOL.

### Electrostatic analysis

**Fig 10:**
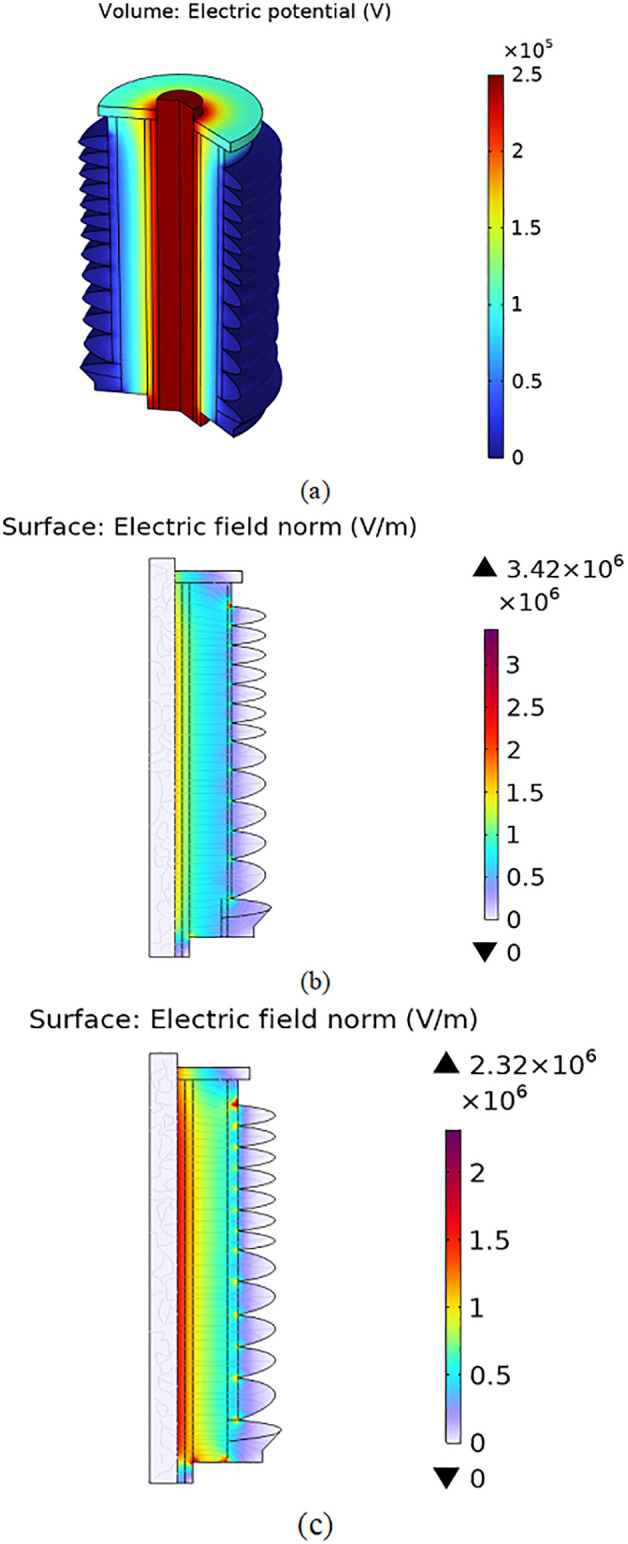
Transformer bushing at: (a) electric potential distribution, healthy; (b) electric field, healthy; (c) Electric field, degraded.

**Fig 11:**
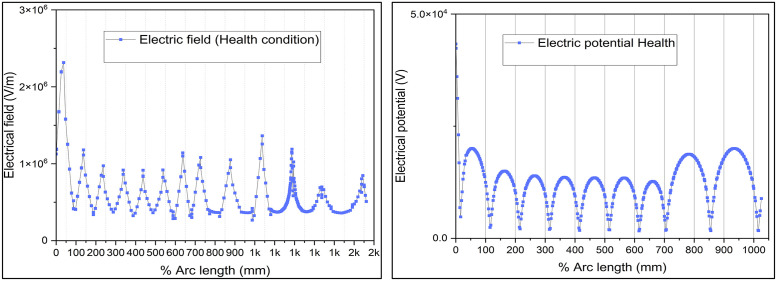
Line graph: (a) Electric field and (b) electric potential grading at bushing health condition.

In this section, the electrostatic analysis of a power transformer bushing is conducted using COMSOL Multiphysics. This study investigates the potential distribution at normal, insulation degradation, and damage conditions. The rated voltage of 11 kV/0.433 kV, 500 kVA is designed for a bushing with a copper core, and the grounding is at the outer porcelain insulator. The study computes the electric potential (V) and electric field (V/m) at the insulator surface. The voltage grading is visualized in Fig 10. The three-dimensional plot in Fig 10a shows the voltage potential grading at a healthy condition. Also, this induces a smooth electric field gradient along the insulation oil, epoxy, and porcelain insulator, as shown in Fig 10b compared to the insulation degradation insulator electric field gradient as shown in Fig 10c. This behavior indicates that in a healthy condition, the electric field controls the localization of electric stress. This potential grading is shown in Fig 11. Fig 11a shows the behavior of the electric field for the bushing at normal operation conditions. The electric field shows a homogeneity peak along the arc length corresponding to the insulator sheds. The field magnitude is controlled within the effective electric field grading at a maximum of 2.3 × 106 V/m. Similarly, the smooth grading for the electric potential along the bushing insulator is shown in Fig 11b. The peak magnitude of the voltage potential is within the safe limits at 2 × 104 V. In this regard, the absence of extensive high or interruption in the field connection indicates the insulation is within the safe limits. The electric field and electric potential profiles in this stage will be used as a reference baseline for future comparison with the degradation and damages intendents results on the transformer bushing.

### Insulation degradation analysis

Transformer bushing insulation degradation caused by electrical, thermal, and environmental stress during long-term operation. the ageing process is due to thermal ageing, partial discharge, and contamination. These activities caused a material change in the dielectric properties of the transformer bushing. There will be an increase in the permittivity and electric conductivity [16]. These changes caused a failure in the transformer bushing in the long term due to insulation degradation and increases in capacitance. The recent studies show weakness in the detection of the early stage of transformer bushing insulation degradation [17]. A motivation in this research is to propose a new approach to modelling and identify insulation changes in the electric field. The thermal caused an insulation material degradation, thus the capacitance value of the transformer bushing decreases.

In this context, the 2D electric field distribution under bushing insulation degradation is illustrated in Fig 10c. The degradation is simulated by modifying the insulation material’s relative permittivity and electrical conductivity. As a result, the electric potential shifts toward the outer insulation sheds, increasing the risk of flashover under severe degradation conditions. The electric field stress along the insulator surface can be quantified using the method described in [18].

The electric field distribution along the insulator is calculated as the negative gradient of the electric potential, as expressed in (4).


$$\begin{array}{c} E=-\nabla V \end{array}$$ (4)

Based on Maxwell’s equations, as follows:


$$\begin{array}{c} \nabla E=\rho /\varepsilon \end{array}$$ (5)

where E is the electric field, V is the applied voltage, ρ is the specific conductivity (μS/m), and ε is the permittivity.

The insulation degradation scenarios are simulated by modifying the insulation material properties, specifically the relative permittivity and electrical conductivity. For the normal condition, the selected materials and their specifications are listed in Table 4. All materials are used with default COMSOL settings, except for deliberate changes in electrical conductivity and relative permittivity, $\varepsilon_{r}=\varepsilon /\varepsilon_{\text{0}}\;$.

**Table 4:** The power transformer selected materials at healthy condition.

Material	Electric conductivity (μS/m)	Relative permittivity ($\varepsilon_{r}$)
Copper	5.998e^7^	1.00
Oil	10e^-9^	2.20
Epoxey	10e^-10^	4.00
**Porcelain**	**1e** ^**-13**^	**10.00**

Under degradation conditions, the porcelain insulation’s electrical conductivity and relative permittivity are modified to reflect the changes in capacitance of the equivalent circuit discussed in the FRA analysis. For this study, a single level of property change is sufficient to examine its effect on the electric potential *(V)* and electric field *(V/m).* The porcelain degradation is represented based on standard values, with the electrical conductivity increased from $\sigma_{\text{0}}=\text{1}\times {\text{1}\text{0}}^{-\text{1}\text{3}}$ S/m to $\sigma_{\text{0},\text{deg}}=\text{1}\times {\text{1}\text{0}}^{-\text{1}\text{1}}$ S/m. The relative permittivity is also increased, typically to $\varepsilon_{r}=\text{1}\text{2}.\text{0}$. In some cases, degradation may additionally reduce the thickness of the outer insulation layer, further affecting the electric field distribution.

**Fig 12:**
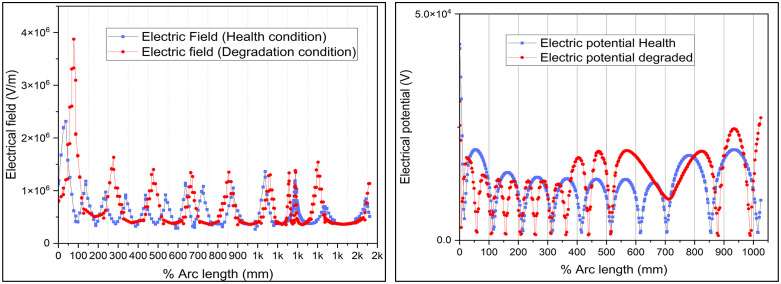
Line graph: (a) electric field and (b) electric potential grading at health vs degradation of bushing insulation.

The results of the electric field and electric potential under bushing insulation degradation are presented in Fig 12. Fig 12a compares the electric field distributions for healthy and degraded conditions. Changes in the insulation material properties lead to an increase in the peak electric field from 2.3 × 10^6^ V/m under healthy conditions to approximately 4 × 10^6^ V/m near the high-voltage region at 100–200 mm. Elevated field peaks are also observed at certain shed locations, such as around 1000 mm.

Fig 12b shows the comparison between the potential distribution on the surface on bushing insulator for healthy and degraded conditions. The potential profile shows higher potential peaks and exhibits drops in voltage at arc lengths above 400 mm. The electric potential at some points reached 3 × 10^4^ V compared to 2 × 10^4^ V for healthy conditions. This indicates that the insulation degradation causes a non-uniform electric stress, which leads to bushing insulation degradation.

### Pollution analysis

The power transformer bushing is exposed to pollution, specifically in outdoor installations. There are various types of pollution censorious such as acidic rain, dust, salt, and humidity. These contaminants will form a conductive layer, which increases the electric field stress on the insulator surface, thus forming a leakage current path [8]. The increase in the possibilities of discharge and forming flashover in advanced stages of pollution. Recent studies have used COMSOL Multiphysics for simulation-relevant transformer bushing insulation ageing, emphasizing the awareness of pollution and condition assessment [19,20].

In this regard, the modelling mechanism was improved by a thin conductive film sprayed on the surface of the bushing insulator and presented in an electrostatic simulation. A seawater-based contamination model represents a realistic conductive pollution condition, specifically:

- The pollution layer conductivity of the sprayed seawater is taken as 45,000 μS/cm representing seawater contamination.
- The pollution severity is defined based on surface coverage: 50% pollution: conductive layer applied randomly to approximately half of the insulator surface area, “partial contamination”. 100% pollution: conductive layer applied to the entire insulator surface (full contamination).

This modeling method allows studying the electric field distribution and surface stress under polluted conditions.

**Fig 13:**
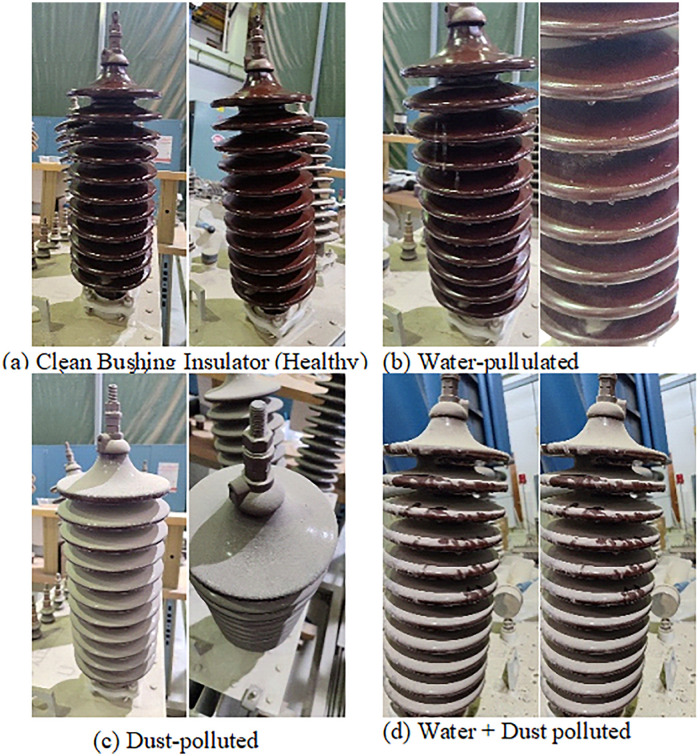
Power transformer bushing showcase in clean and polluted circumstances.

**Fig 14:**
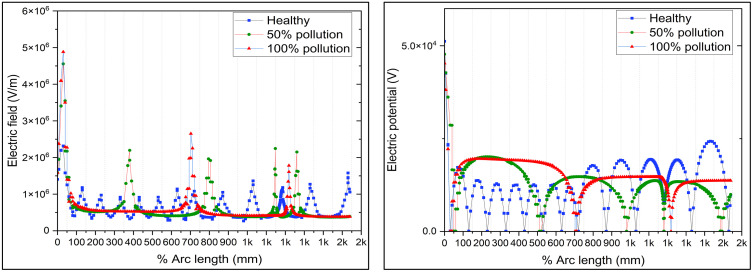
Line graph: (a) Electric field, (b) Electric potential on the bushing surface at normal vs pollution conditions.

The clean bushing is shown in Fig 13a. Hence, the polluted insulators are in Fig 13b, Fig 13c, and Fig 13d. There is water on the insulator surface, dust, and water with dust, respectively. The pollution phenomena form with the time and contamination of dust, plus the humidity forms the pollution layer. The simulation of this bushing pollution is performed by adding a thin, conductive, and resistive layer of contamination (ESDD) particles on the surface of the bushing insulators. The simulations evaluate the resulting electric field distortion and leakage currents, with the results presented in Fig 14.

Figs 14a and 14b illustrate the effect of pollution on the electric field and electric potential distributions along the transformer bushing surface. Fig 14a shows that the electric field magnitude increases at locations near the high-voltage end (around 50 mm) and at certain arc lengths, such as 700 mm and 1000 mm. At the high-voltage region, the peak electric field reaches 4.5 × 10^6^V/m for 50% pollution and 5 × 10^6^V/m for 100% pollution. Fig 14b presents the corresponding electric potential distributions for healthy and polluted conditions. Pollution causes the potential profile to become non-uniform, with steeper voltage drops along the insulator surface. The distortion of the electric field is more pronounced at 100% pollution compared to 50%. Near the high-voltage point, the electric potential decreases from 5.1 × 10^4^V (healthy) to 4.8 × 10^4^V (50% pollution) and 4.5 × 10^4^V (100% pollution). These results indicate that pollution forms a conductive layer on the insulator surface, creating non-uniform paths for leakage currents from the high-voltage conductor to ground. Consequently, the likelihood of partial discharges and flashover increases with the severity of contamination.

### Insulator damage analysis

**Fig 15:**
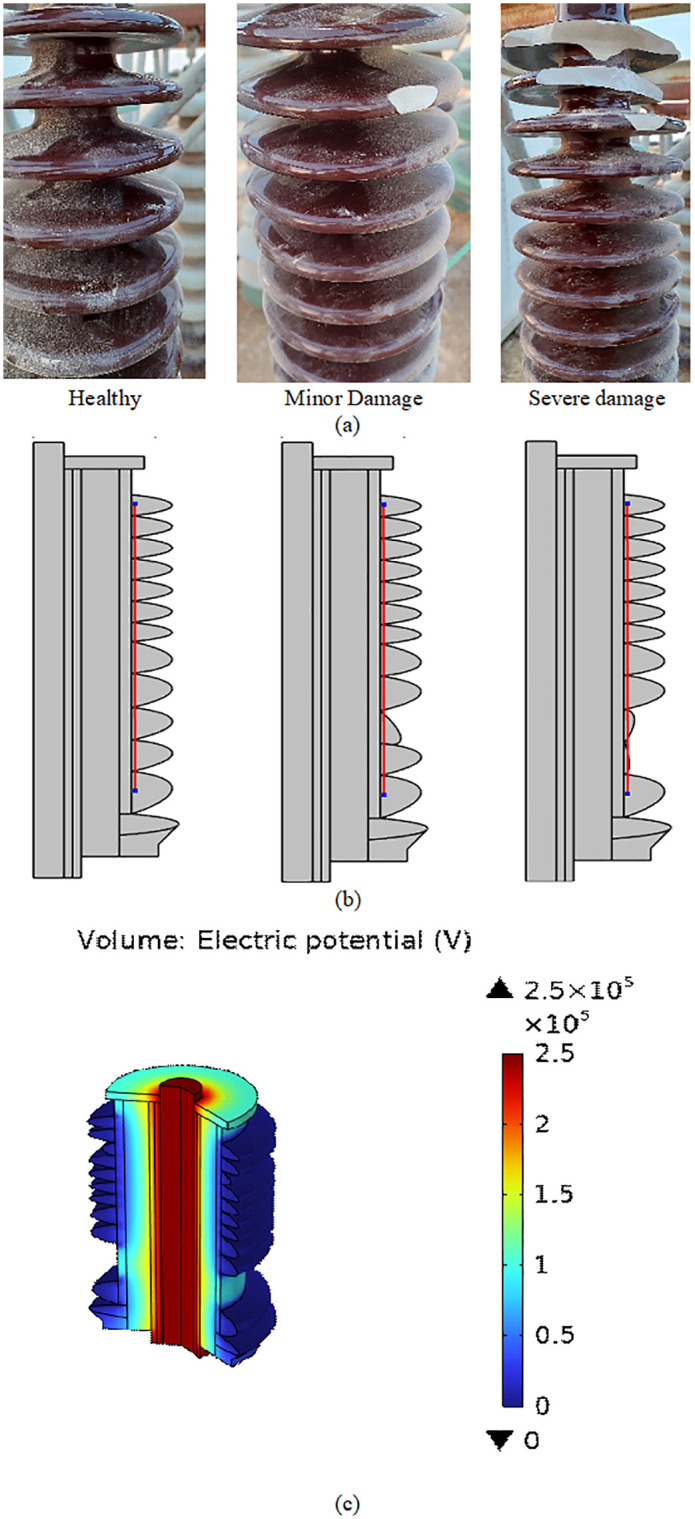
Bushing damage simulation (a) 2D damage boundaries, (b) Transformer bushing damage showcase, (c) 3D boundaries condition of the simulation.

Transformer bushing insulation damage can result from impacts by falling objects in harsh environments, loose fittings, improper terminal tightening, mishandling during maintenance, or flashover events [21]. Such damage alters the physical structure of the insulator, which can be represented in COMSOL simulations. Damage may manifest as dimensional changes or, in severe cases, complete fracture of the insulator, as illustrated in Fig 15a for a healthy, minor-damage, and severe-damage insulator. Minor damage typically involves chipping at the tip of an outer shed, whereas severe damage affects one or more sides of the shed. To simulate bushing damage, the geometry of the sheds is modified to reflect these dimensional changes. The approximated simulation model is shown in Fig 15b, with the corresponding 3D electric potential distribution presented in Fig 15c.

**Fig 16:**
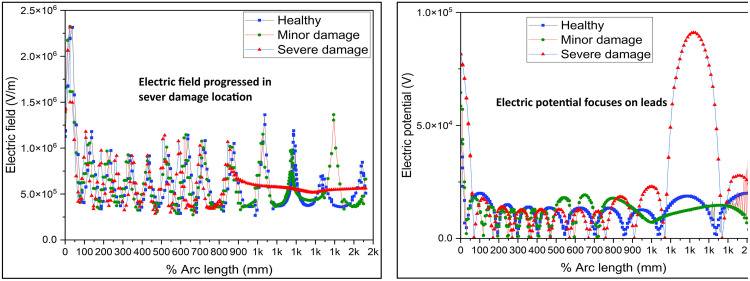
Line graph: (a) Electric field, (b) Electric potential for healthy and damaged conditions.

The electric field results for bushing insulator damage are presented in Fig 16a. Minor damage produces a moderate increase in the electric field magnitude, particularly at the modelled damaged shed around 1000 mm, where the field rises from approximately 5 × 10^5^ V/m to 1.25 × 10^6^ V/m. In the case of severe damage, the electric field is significantly distorted along the bushing surface, especially above 1000 mm. Fig 16b shows the corresponding electric potential distributions. Minor damage causes noticeable distortions and localized increases in potential, while severe damage leads to substantial increases, with peak potentials approaching 9 × 10^4^ V. Severe damage disrupts the intended capacitive grading of the bushing sheds, resulting in elevated electrical stress and accelerated insulation ageing.

## Bushing failure mapping and discussion

In this paper, both electrical circuit modelling and electrostatic analysis are employed to evaluate and map insulation failures in power transformer bushings. This study demonstrates the correlation between bushing failures, capacitance, and its dielectric properties, such as the structure of insulation parts and the insulation materials, like electric conductivity, relative permittivity. The application of this paper can be highlighted as the early detection of the transformer bushing insulation degradation, and the physical interpretation of the FRA deviations between the normal and electrical stress in transformer bushings. Also, it is for the classification of different failures in transformer bushings and condition-based maintenance management.

Table 5 shows the maximum peak point at which the electric field and electric potential approach along the arc length. Notably, the bushing failures increase the peak values. Fundamentally, the bushing insulation degraded, resulting in changes in the insulation material properties. The dielectric primitivity changes, and the electrical conductivity increases. Therefore, the effective capacitance within the insulation material decreases as discussed in electrical approach analysis section.

**Table 5:** Electric field and electric potential maximum peak point at arc length for health, degradation, pollution, and damage conditions.

Bushing condition	Electric field (10^6^ V/m)	Electric Potential (10^4^ V)
Healthy	2.30	1.80
Degraded insulator	4.00	3.00
Polluted shed 50%	4.60	2.50
Polluted shed 100%	5.20	3.00
Minor shed damaged	2.40	3.00
Severe shed damage	2.50	9.00

For comparison, Tables 1, 2, and 5 can be used to study the behaviour of the electric circuit capacitance and electrostatic (electric field and electric potential) when the transformer bushing gets degraded. The relationship between the two models is established to study the bulk insulation degradation and the surface-related effects on the transformer bushing. Table 1 presents the power transformer equivalent circuit parameters under healthy conditions, while Table 2 defines the degradation levels through progressive changes in bushing element capacitance. In the electrostatic model, Table 5 presents the simulated electric field and electric potential under normal and pollution conditions using a seawater layer with a conductivity of 45,000 μS/cm and surface cover of 50% and 100%.

In the electrostatic simulation, pollution is represented by a conductive surface layer applied to the transformer bushing insulator. An assumption conductivity of 45,000 μS/cm, corresponding to seawater conductivity, is adopted to represent a severe coastal contamination scenario under wet conditions. The purpose of this assumption is to investigate the worst-case influence of highly conductive surface pollution on electric field distribution and voltage grading.

As the transformer bushing capacitance deteriorated from 10% up to 50%, it is observed that the FRA signature shifts toward lower frequencies and increased damping magnitude. This indicates a deterioration of the bushing insulation system. The electrostatic simulation captures surface-related degradation on 50% and 100%, where the electric field magnitude increases significantly, and the electric potential distribution becomes increasingly non-uniform. These progressive changes in electric field and electric potential results have become more at 100% compared to 50%.

Tables 1, 2, and 5 will be used to analyze the behavior of transformer bushing insulation under different degradation scenarios. This analysis offers a comprehensive understanding of bushing degradation mechanisms and proposes the failure mapping framework in Table 6.

**Table 6:** Transformer bushing physics-based failure mapping.

Bushing condition		Physical condition	Capacitance variation	FRA behavior	Electrostatic behavior
Healthy		The dielectric properties of the material are stable	0%	Bushing FRA baseline	Smooth electric field and potential grading along the arc length
Degradation	early	Moisture ingress and ageing	< 10%	Small resonance shift towards mid-frequency	Small electric field increases at the shed edges
	moderate	Insulation material, primitivity, and losses increase	>20%	Resonance shift towards mid-frequency + magnitude reduction	Noticeable electric field and potential increases at the HV point (>30%).
	advanced	Moisture on the insulator and severe ageing	30%	Resonance shift towards mid-frequency + high damping	
Pollution	50%	Insulator surface conductivity increases	>30%	Shifting resonance more towards lower frequencies	Elevated surface electric field and distorted electric potential
	100%	A heavy conductive pollution layer over the insulator	40%	Shifting resonance more towards lower frequencies + magnitude drop	Very high distorted electric field at the shed arc length, and a peak at near HV point (>60%). flashover possibilities occur.
Damage	minor	Creak or erosion	20%	Small deviation at high frequency	Local electric field increases at the damaged shed. 30% increases in the peak at arc length.
	severe	Tracking carbonization	>50%	Resonance drops at low and mid-frequency regions	Extreme electric field increases and electric potential at the damaged shed. 100% increases in peak at arc length

By comparing the FRA results with the electrostatic simulations, a failure-mapping framework for the transformer bushing is presented in Table 6. It shows that small capacitance decreases (<10%) indicate the early stages of insulation degradation, causing a shift in the FRA response toward mid-frequency ranges. For capacitance decreases of 20% – 30%, corresponding to developing insulator pollution, the FRA signature shifts further toward lower frequencies. Capacitance decreases exceeding 40% represent severe insulation damage, resulting in pronounced damping of the FRA signature across both low- and mid-frequency regions.

## Conclusion

Transformer bushing insulation failure is typically associated with ageing and thermal stress. This paper proposes a method for mapping such failures using FRA and electrostatic simulations. The FRA results indicate that even a small reduction in bushing capacitance (≈10%) causes resonance frequencies to shift from high to mid-frequency ranges. As the capacitance decreases further (30% – 50%), the resonance peaks shift toward lower frequencies, accompanied by a reduction in magnitude. The deviation between the healthy and faulty bushing conditions is quantified using the correlation coefficient (CC), which decreases to 0.76–0.83 under severe fault scenarios. Additionally, the radar (spider) plots provide a clear visualization of insulation degradation across different FRA frequency regions. The electrostatic simulations illustrate the electric field distribution and stress along the transformer bushing. Under healthy conditions, both the electric field and potential exhibit smooth grading along the bushing arc length. Insulation degradation, pollution, or mechanical damage introduces steeper voltage gradients. Near the high-voltage end, the electric field and potential peaks increase by over 60% in the presence of degradation or pollution compared to healthy conditions. Minor damage produces modest increases, whereas severe damage can elevate the peaks up to approximately 98%, particularly along the shed arc lengths. A clear correlation exists between the FRA signatures, electrostatic analysis results, and the underlying degradation mechanisms in transformer bushings. This combined approach enhances the understanding and assessment of bushing condition and provides a framework for identifying specific failure types using both FRA and electrostatic simulations. At this stage, the transformer bushing is measured in a healthy condition. Future work could extend this methodology to the entire insulation system, enabling a more comprehensive and reliable diagnostic framework for power transformer bushings.
